# Three-Dimensional Evaluation of Marginal Gap in Prefabricated Zirconia and 3D-Printed Pediatric Crowns

**DOI:** 10.3390/ma19163400

**Published:** 2026-08-11

**Authors:** Büşra Karaağaç Eskibağlar, İrem İpek, Ayşe Rençber Kızılkaya

**Affiliations:** 1 Department of Pediatric Dentistry, Faculty of Dentistry, Fırat University, 23119 Elazığ, Turkey; 2 Department of Prosthodontics, Faculty of Dentistry, Fırat University, 23119 Elazığ, Turkey

**Keywords:** marginal gap, 3D printing, pediatric crowns, prefabricated zirconia crowns, Geomagic Control X

## Abstract

**Objective:** This study aimed to compare the marginal gap values of prefabricated zirconia and resin crowns fabricated using different three-dimensional (3D) printing technologies for primary molars using a 3D digital analysis method. **Materials and Methods:** Forty crowns (n = 10/group) were evaluated, including prefabricated zirconia crowns (NuSmile) and three resin crown systems fabricated using different additive manufacturing technologies: Crowntec (Saremco, DLP), Permanent Crown Resin (Formlabs, SLA), and Graphy TC-80DP (MSLA). All restorations were cemented with self-adhesive resin cement. The specimens were scanned before and after cementation, and the obtained STL datasets were superimposed using the Geomagic Control X software. Marginal gap values were calculated using three-dimensional (3D) digital analysis. Data were analyzed using one-way ANOVA and Games–Howell post hoc tests (α = 0.05). **Results:** The mean marginal gap values differed among the groups. The lowest marginal gap value was observed in the Formlabs group (113.22 ± 1.94 µm), followed by that of the Saremco group (118.54 ± 1.58 µm). Higher marginal gap values were recorded in the Graphy group (134.23 ± 2.16 µm), whereas the highest value was observed in the prefabricated zirconia crown group (163.14 ± 3.95 µm). Statistically significant differences were observed among all groups (*p* < 0.001), with all 3D-printed resin crown systems exhibiting lower marginal gap values than the prefabricated zirconia crowns. **Conclusions:** Within the limitations of this in vitro study, 3D-printed resin crowns exhibited superior marginal adaptation compared to prefabricated zirconia crowns. Among the evaluated materials, the SLA-fabricated Formlabs crowns exhibited the lowest marginal gap values. All tested resin crown systems demonstrated marginal gap values within clinically acceptable limits.

## 1. Introduction

Dental caries is one of the most prevalent chronic infectious diseases in childhood, exhibiting a high prevalence in primary dentition during early childhood and adversely affecting oral health from both functional and esthetic perspectives [1]. The American Academy of Pediatric Dentistry (AAPD) recommends full-coverage crowns for primary teeth with extensive carious lesions or those treated with pulp therapy, emphasizing that such restorations are one of the most effective treatment options for preserving structural integrity and preventing recurrent caries [2].

Stainless steel crowns have long been considered the standard treatment option for primary teeth with extensive structural loss owing to their durability, high clinical success rates, and ease of use [3,4]. However, the metallic appearance of these restorations may negatively affect parental and child satisfaction from an esthetic perspective [5]. To overcome this limitation, prefabricated zirconia crowns (PZCs) have been developed and gained popularity owing to their superior mechanical strength, biocompatibility, and tooth-like esthetic properties [6]. In addition, their monolithic structure provides enhanced resistance to fracture and discoloration, and their smooth surface characteristics contribute to periodontal health by reducing plaque accumulation [7,8]. Nevertheless, the need for more extensive tooth preparation and limited adaptability during clinical adjustment due to low flexibility remain important disadvantages of these restorations [9].

The emergence of three-dimensional (3D) printing technology has significantly expanded clinical possibilities, enabling clinicians to provide highly accurate, rapid, customizable, and esthetically optimized treatment options [10]. Dental restorations can be fabricated directly or indirectly using resin-based additive manufacturing technologies [11]. Owing to advantages such as the need for more conservative tooth preparation, ease of adaptation, shorter fabrication times, and reduced chairside time for patients, these technologies offer an alternative treatment approach in pediatric dentistry [12].

Although previous studies have compared PZCs with digitally fabricated or 3D-printed crowns for primary teeth, limited information is available regarding whether different 3D-printing systems provide comparable marginal gap outcomes under standardized experimental conditions. Evaluating DLP, SLA, and MSLA based crown fabrication workflows within the same experimental design may provide clinically relevant information because 3D-printed pediatric crowns should not necessarily be considered a homogeneous restorative category in terms of marginal fit. Therefore, to the best of our knowledge, no previous study has directly compared the marginal gap values of PZCs and 3D-printed resin crowns fabricated using DLP, SLA, and MSLA based systems using a 3D digital analysis method. The aim of this study was to compare the marginal gap values of PZCs and 3D-printed resin crowns fabricated using different 3D-printing systems by means of a 3D digital analysis method. The null hypothesis of this study was that there would be no statistically significant difference in marginal gap values between PZCs and 3D-printed resin crowns fabricated using different 3D-printing systems.

## 2. Materials and Methods

Sample size calculation was performed using G*Power software (Version 3.1.9.7; Heinrich Heine University, Düsseldorf, Germany). An a priori power analysis was conducted using the F-test family (one-way ANOVA: fixed effects, omnibus, one-way) assuming four independent groups. The calculation was based on a significance level of 0.05, a statistical power of 79.8%, and a large effect size (f = 0.55). The selected effect size was determined based on Cohen’s recommendations for a large effect and considering the magnitude of the differences reported in previous in vitro studies evaluating the marginal adaptation of pediatric crowns [3,13].

The study protocol was approved by the Non-Interventional Ethics Committee of Fırat University (2025/11-28). In this study, three different photopolymer resin-based crown materials [Crowntec (Saremco Dental AG, Rebstein, Switzerland), Permanent Crown Resin (Formlabs Inc., Somerville, MA, USA), and Graphy TC-80DP (Graphy Inc., Seoul, Republic of Korea)] and one PZC (NuSmile, Houston, TX, USA) were used. In addition, one mandibular second primary molar, extracted for reasons unrelated to this study, was included as a reference tooth specimen. Written informed consent for the extraction procedure was obtained from the patient’s parent or legal guardian prior to tooth extraction. The extracted tooth was anonymized before inclusion in the study.

The mandibular second primary molar was prepared according to the preparation protocol recommended for PZCs. The prepared tooth was scanned using an intraoral scanner (CEREC Omnicam, Dentsply Sirona, Bensheim, Germany), and the resulting digital model was saved in STL format. The STL model was then duplicated 40 times (n = 10 per group) using a 3D printer UBEE (UNIZ Technology LLC, Shanghai, China). This approach minimized potential variations during the fabrication and evaluation processes by ensuring that all crowns were produced and tested on tooth models with identical anatomical characteristics. A CAD software program (DentalCAD, Version 3.0 Galway; exocad GmbH, Darmstadt, Germany) was used to design the primary molar crown based on the STL data. The cement space was set to 50 μm.

### 2.1. Fabrication of Restorations

#### 2.1.1. Crowntec Resin Group (Digital Light Processing 3D Printer)

Ten restorations were fabricated using Crowntec resin (Saremco Dental AG, Rebstein, Switzerland) at a printing angle of 0° and layer thickness of 50 μm. The restorations were manufactured according to the manufacturer’s recommendations using an Asiga MAX UV 3D printer (Asiga, Alexandria, NSW, Australia) based on Digital Light Processing (DLP) technology. The fabricated specimens were cleaned in 99% isopropyl alcohol for 1 min, followed by post-polymerization using a VALO LED light-curing unit (Ultradent Products, Inc., South Jordan, UT, USA) for 6 min.

#### 2.1.2. Permanent Crown Resin Group (Stereolithography 3D Printer)

Ten restorations were fabricated using Permanent Crown Resin (Formlabs Inc., Somerville, MA, USA) using a Form 3B+ 3D printer (Formlabs Inc., Somerville, MA, USA) at a printing angle of 0° and a layer thickness of 50 μm. After fabrication, the specimens were cleaned in 99% isopropyl alcohol for 3 min and subsequently post-polymerized in a Form Cure unit (Formlabs Inc., Somerville, MA, USA) at 60 °C for 20 min.

#### 2.1.3. Graphy Resin Group (Masked Stereolithography Apparatus 3D Printer)

Ten restorations were fabricated using Graphy TC-80DP resin (Graphy Inc., Seoul, Republic of Korea) at a printing angle of 0° and layer thickness of 50 μm. The restorations were fabricated using a UBEE 3D printer based on masked stereolithography (MSLA) technology (UNIZ Technology LLC, Shanghai, China). Following fabrication, the specimens were cleaned in 99% isopropyl alcohol according to the manufacturer’s instructions and subsequently post-polymerized using a Tera Harz Cure unit (Graphy Inc., Seoul, Republic of Korea) under nitrogen mode (Level 1) at 30 °C for 15 min. The curing unit employed a UV-LED light source with a wavelength of 405 nm, a power output of 200 W, and irradiance of 1000 mW/cm^2^.

#### 2.1.4. Prefabricated Zirconia Crown Group

No fabrication procedure was performed on the PZCs (NuSmile, Houston, TX, USA). Instead, the appropriate crown size (E3R) was selected for the tooth model and applied as per the manufacturer’s instructions.

### 2.2. Cementation Procedure

**Figure 1:**
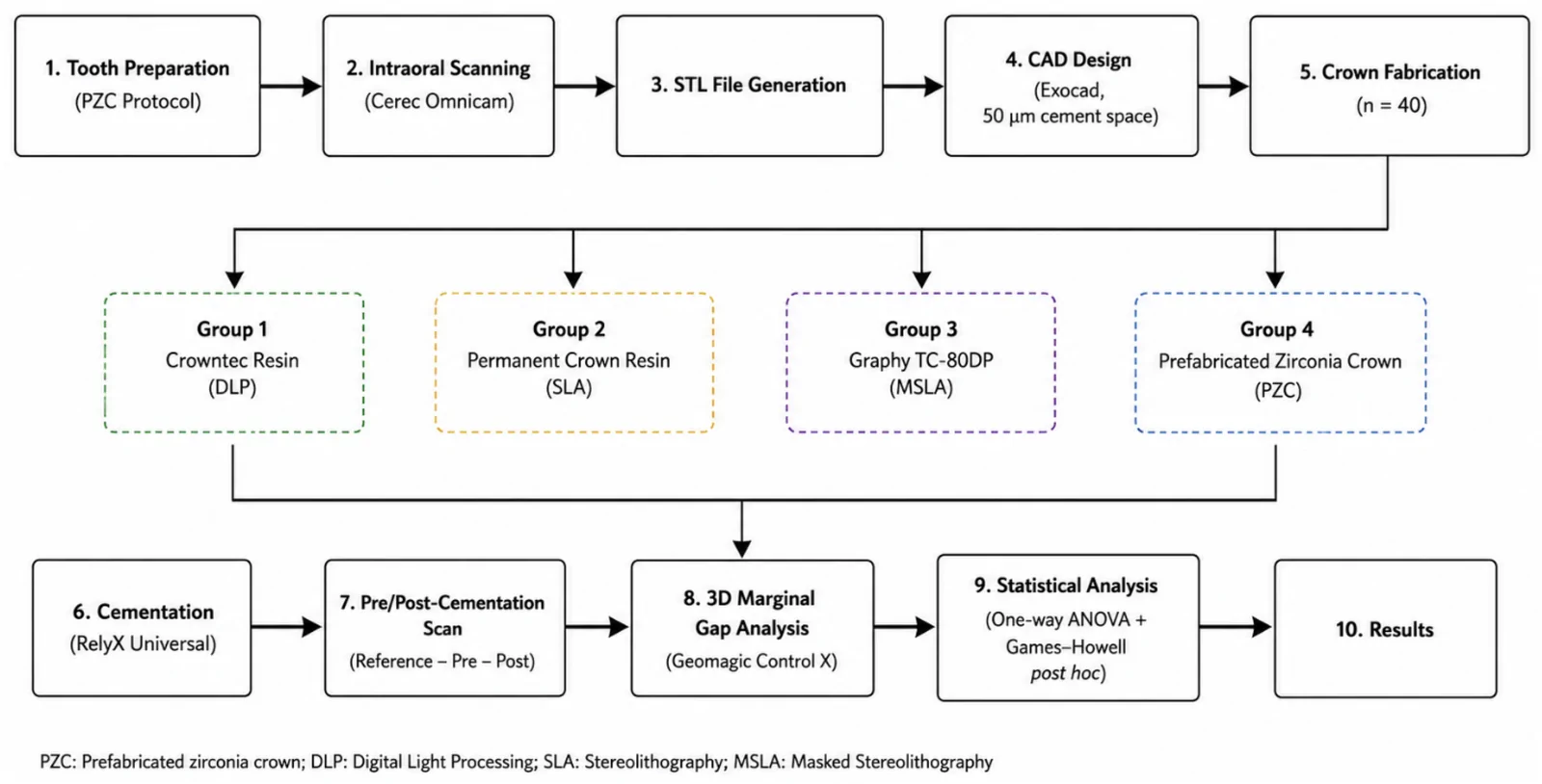
Flowchart of the study methodology.

A self-adhesive resin cement (RelyX Universal Resin Cement; 3M ESPE, St. Paul, MN, USA) was used to cement all crown groups. Each crown was initially seated on the corresponding tooth model using finger pressure to ensure proper positioning. Subsequently, a standardized vertical load of 5 kg (approximately 50 N) was applied manually for 5 min. No mechanical loading device was used during the cementation procedure, and the same loading protocol was applied consistently to all specimens. After removing the excess cement, light polymerization was performed for 10 s on the buccal, lingual, and occlusal surfaces. The overall study workflow, including crown fabrication, cementation, scanning, and marginal gap analysis procedures, is illustrated in Figure 1.

### 2.3. Marginal Gap Analysis

Prior to cementation, the 3D-printed tooth models and all crown restorations were scanned using a digital scanner (Solutionix C500; Medit Corp., Seoul, Republic of Korea), and the acquired data were saved in STL format. These scans were performed to establish a pre-cementation reference dataset. Following the completion of the cementation procedure, all specimens were rescanned and saved in STL format. The STL datasets obtained before and after cementation were comparatively analyzed using Geomagic Control X software (Version 2022.1.0; 3D Systems, Rock Hill, SC, USA). The datasets were initially registered using the Initial Alignment function and subsequently refined using the Best Fit Alignment algorithm. The alignment procedure was performed using a software tolerance setting of ±0.02 mm. Before the measurements, the evaluator reviewed the measurement protocol, predefined anatomical landmarks, and standardized the measurement procedure to ensure consistent measurements throughout the analysis. A circular reference geometry was created at the center of each specimen to standardize the measurement’s orientation. Based on this reference geometry, four cross-sectional planes were generated at 0°, 45°, 90°, and 135° angles. Two point-based linear marginal gap measurements were obtained from each cross-section using the Comparison Point tool. To improve measurement reliability, all measurements were repeated three times by the same blinded evaluator during the same measurement session using the same standardized protocol, and the arithmetic mean of the repeated measurements was used for statistical analysis. Intra-examiner reliability was assessed using the intraclass correlation coefficient (ICC) based on a two-way mixed-effects model with absolute agreement.

### 2.4. Statistical Analysis

Statistical analyses were performed using SPSS software (Statistical Package for the Social Sciences, Version 25.0; IBM Corp., Armonk, NY, USA). The normality of the data distribution was assessed using the Shapiro–Wilk test. After confirming normality, differences among the four groups were analyzed using one-way analysis of variance. Because the homogeneity of variance assumption was not satisfied, Games–Howell post hoc comparisons were performed. The effect size was expressed using eta squared $\left(\eta^{2}\right)$ with its 95% confidence interval. Statistical significance was set at *p* < 0.05.

## 3. Results

The intra-examiner reliability was high for the averaged repeated measurements (ICC = 0.941; 95% CI: 0.631–0.987). The mean (SD) values for all subgroups in µm are listed in Table 1. The mean marginal gap values differed between the groups. The lowest marginal gap value was observed in the Formlabs group (113.22 ± 1.94 µm), followed by that of the Saremco group (118.54 ± 1.58 µm). Higher marginal gap values were observed for the Graphy group (134.23 ± 2.16 µm), while the highest value was observed in the PZC group (163.14 ± 3.95 µm). Overall, the 3D-printed resin groups demonstrated lower marginal gap values than the PZC group.

One-way ANOVA revealed a statistically significant difference among the four groups (F(3,36) = 758.072, *p* < 0.001, η^2^ = 0.98 [0.97–0.99]). Games–Howell post hoc analysis demonstrated that all groups differed significantly from each other (*p* < 0.001). The lowest marginal gap value was observed in the Formlabs group (113.22 ± 1.94 µm), followed by the Saremco group (118.54 ± 1.58 µm), the Graphy group (134.23 ± 2.16 µm), and the PZC group (163.14 ± 3.95 µm).

**Figure 2:**
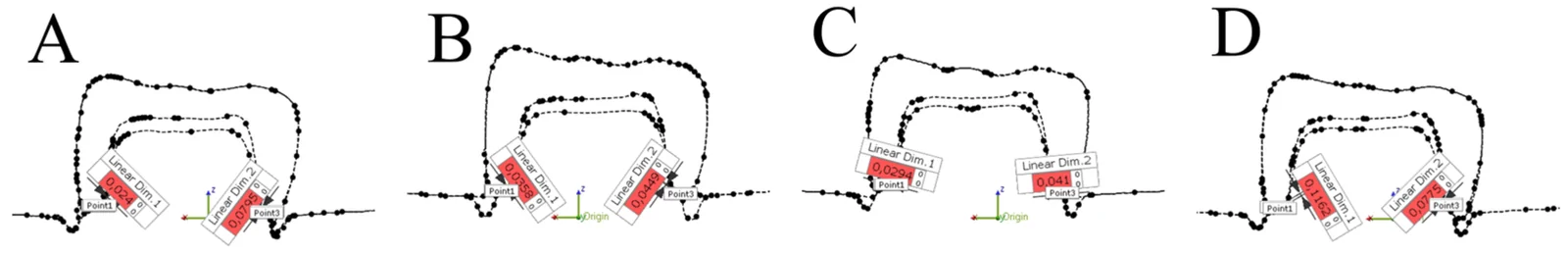
Representative Geomagic Control X cross-sectional images of the evaluated crown systems: (**A**) Formlabs; (**B**) Saremco; (**C**) Graphy; and (**D**) PZC.

**Figure 3:**
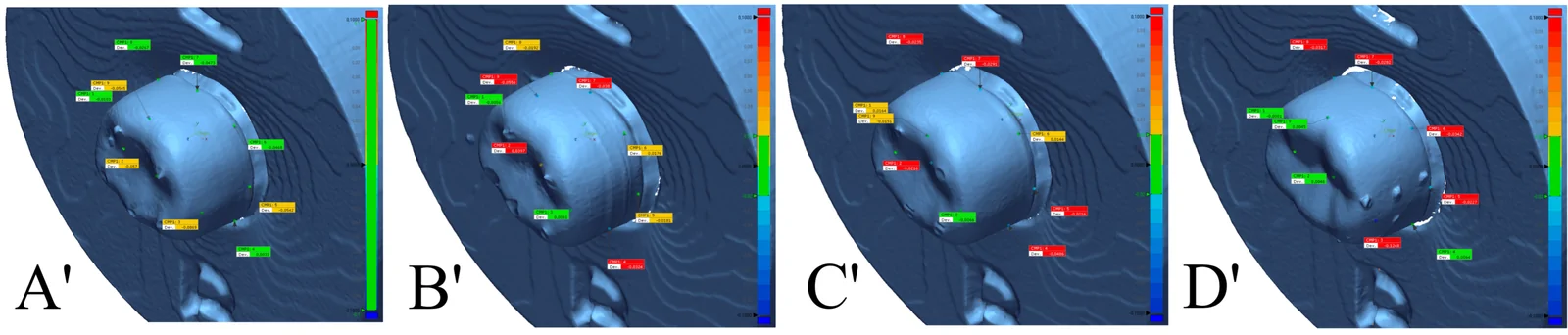
Geomagic Control X–generated representative color deviation maps of the evaluated crown systems: (**A′**) Formlabs, (**B′**) Saremco, (**C′**) Graphy, and (**D′**) PZC.

Representative Geomagic Control X cross-sectional images and color deviation maps of the evaluated crown systems are presented in Figure 2 and Figure 3.

## 4. Discussion

In this study, the marginal gap values of PZCs and 3D-printed resin crowns fabricated using different manufacturing technologies for primary second molars were compared, and the effects of both the restoration type and production parameters on these values were evaluated. The findings demonstrated that the type of restoration had a statistically significant effect on the marginal gap values. Therefore, the null hypothesis was rejected.

The adoption of digital technologies and computer-aided design/computer-aided manufacturing (CAD/CAM) systems has significantly advanced restorative treatment procedures. Additive manufacturing technologies produce structures through layer-by-layer deposition of small volumes of materials [14,15]. Compared with conventional CAD/CAM milling techniques, 3D printing offers several advantages, including reduced fabrication time, decreased material waste, and the ability to create customized designs for dental prostheses. Consequently, it is considered a cost-effective alternative for fabricating dental restorations [16]. In line with these advantages, the present study evaluated the marginal gaps of 3D-printed resin crowns fabricated using different additive manufacturing techniques, namely DLP, SLA, and MSLA.

The marginal gap is influenced not only by the manufacturing technology and material composition but also by the cementation protocol. In the present study, the same self-adhesive resin cement was used in all the groups, and the pressure applied during cementation was standardized. Thus, the potential influence of cementation-related variables on the observed intergroup differences was minimized. In addition, a cement space of 50 μm was applied during the digital design phase for all groups. Therefore, the observed differences are more likely to be related to manufacturing technology and material characteristics than to variations in cement thickness.

In the present study, Geomagic Control X software was used because it provides an objective and reproducible evaluation protocol that reduces operator dependency in marginal gap measurements through its ability to accurately align high-resolution point cloud data with a reference model. Geomagic Control X software has been reported to offer high methodological reliability in the accuracy analysis of dental models, as evidenced by its low root mean square (RMS) error values [17]. Therefore, the marginal gap values were evaluated using the Geomagic Control X software, a 3D digital analysis method.

When the marginal gap results of the present study were evaluated, the 3D-printed resin crowns fabricated using 3D printing technology exhibited lower marginal gap values than the PZCs (163.14 ± 3.95). This finding may be attributed to the ability of digitally manufactured restorations to be designed according to the specific geometry of the tooth preparation and provide a customized fit. In contrast, because PZCs are manufactured in standardized sizes and morphologies, they may not fully adapt to the prepared tooth surface, resulting in increased marginal gap values. In a study conducted by Salman et al. [18], the marginal gap values of PZCs were evaluated, and these restorations exhibited higher marginal discrepancies. Similarly, Aktaş et al. [3] compared PZCs with CAD/CAM-milled and 3D-printed restorations and reported that PZCs demonstrated the highest marginal gap values. Similarly, Elnagar et al. [13] evaluated the marginal adaptation of 3D-printed microfilled hybrid resin crowns and PZCs in pulpotomized primary teeth and reported that PZCs exhibited greater marginal gaps.

In DLP systems, the simultaneous polymerization of an entire layer through projection can provide more homogeneous curing, thereby improving dimensional accuracy. In contrast, the point-by-point polymerization process performed using lasers in SLA systems may lead to manufacturing-related variations. In MSLA or LCD-based systems, the pixel-based distribution of light may affect the edge accuracy [19]. This finding indicates that marginal adaptation is influenced not only by the material itself but also by the manufacturing technology employed for it. However, the performance of manufacturing systems is also dependent on the physical and chemical properties of the resin materials. The resins used in 3D printing systems are composed of monomeric acrylic esters with varying filler contents and polymer network characteristics. Resin formulation and filler content may affect the mechanical properties, polymerization shrinkage, and dimensional stability of the restoration [20,21,22]. In addition, processing parameters such as manufacturer-specific washing times, UV light exposure duration, and curing temperature may influence the accuracy of the final restoration [20,23,24]. Accordingly, the 3D-printed resin crowns used in the present study were fabricated using permanent resin materials recommended by the manufacturers for each 3D printing system, thereby minimizing the influence of process-related variables and ensuring standardization. Therefore, the observed differences may be attributed not only to the manufacturing technology but also to the differences in material properties. However, because each manufacturing technology evaluated in the present study was represented by a single commercially available material–printer system, the observed differences cannot be attributed solely to the printing technology itself. Resin formulation, printer-specific calibration, proprietary manufacturing workflows, and postcuring protocols may also have contributed to the observed marginal gap values. Therefore, the findings should be interpreted as reflecting the combined effects of the manufacturing system and the material rather than the printing technology alone.

Wadhwani et al. [25] compared the marginal adaptation and surface roughness of 3D temporary restorations fabricated using SLA and DLP technologies and reported that the restorations produced with SLA exhibited lower marginal gap values and superior marginal adaptation compared with those fabricated using DLP. The authors suggested that this finding may be attributed to the accuracy losses associated with the simultaneous polymerization of an entire layer in DLP systems, whereas the point-by-point polymerization achieved by a UV laser in SLA technology may allow more precise fabrication, resulting in lower marginal gap values. Similarly, Abdelmohsen et al. [26] evaluated the marginal and internal fit of provisional fixed dental prostheses fabricated using SLA and DLP technologies and reported that the SLA group exhibited the lowest marginal and internal gap values. The authors further stated that the longer post-polymerization time and higher curing temperature applied to SLA restorations may increase the degree of polymerization, thereby influencing their dimensional accuracy. Farag et al. [27] evaluated the marginal adaptation of 3D-printed crowns and reported that crowns fabricated using SLA exhibited lower marginal gap values than those produced using DLP technology. Consistent with the findings of previous studies, the lowest marginal gap value in the present study was observed in the SLA-fabricated Formlabs group (113.22 ± 1.94), whereas the highest marginal gap value was recorded in the MSLA-fabricated Graphy group (134.23 ± 2.16). Although statistically significant differences were observed among the 3D-printed crown groups, the Graphy group exhibited the highest marginal gap values among the printed materials. This finding may be attributed to excessive polymerization of the material during fabrication due to light scattering, resulting in greater material hardening than intended [28].

According to Belser et al. [29], a marginal gap value of up to 120 μm is considered clinically acceptable, whereas Beuer et al. [30] reported a threshold of 100–150 μm. In the present study, all the 3D-printed resin crown materials demonstrated marginal gap values within the clinically acceptable ranges reported in the literature. Although statistically significant differences were observed among the 3D-printed crown groups, all measured values remained within these clinically acceptable limits. In particular, the approximately 5 μm difference between the Saremco (118.54 μm) and Formlabs (113.22 μm) groups, although statistically significant, is unlikely to be clinically meaningful. Therefore, the lower marginal gap value observed in the Formlabs group should be interpreted as reflecting a difference in marginal adaptation under the present experimental conditions rather than as clear evidence of superior clinical performance. Long-term clinical studies are required to determine whether these statistically significant differences translate into clinically relevant outcomes. Adequate marginal adaptation is essential for maintaining mechanical stability, durability, and the health of the surrounding tissues. An increase in the marginal gap may elevate the risk of cement dissolution resulting from physical fatigue and chemical corrosion, thereby increasing the likelihood of microleakage. Consequently, this may promote subgingival biofilm accumulation and bacterial colonization, potentially leading to secondary caries and periodontal inflammation [31]. Therefore, it is important to determine the effects of different manufacturing technologies and material types on marginal gap values to evaluate the long-term clinical performance of the crowns.

Although the findings of the present study are promising, several limitations must be acknowledged. First, the study was conducted under in vitro conditions and did not incorporate dynamic factors that may influence the performance of restorations in the oral environment. Additionally, marginal gap measurements were performed immediately after cementation, and no artificial aging procedures, such as thermal cycling or mechanical fatigue, were applied. Therefore, the potential effects of polymerization stress, water sorption, thermal fluctuations, and cyclic mechanical loading on the long-term marginal integrity of the restorations could not be evaluated. Second, all crowns were fabricated and evaluated using duplicated models derived from a single prepared primary molar. Although this approach minimized preparation-related variability and enabled standardized comparisons among the experimental groups, it did not account for the natural anatomical variations observed among primary molars, which may limit the external validity of the findings. In addition, only 3D-printed resin crowns and PZCs were evaluated in this study. Therefore, the findings may not be directly applicable to other crown systems or restorative materials used. Future studies involving multiple tooth preparations, artificial aging protocols, different crown systems and materials, various scanner systems and manufacturing technologies under clinically relevant conditions will further enhance the clinical relevance and generalizability of the results.

## 5. Conclusions

Within the limitations of this in vitro study, 3D-printed resin crowns demonstrated better marginal adaptation than PZCs. Among the tested materials, the SLA-fabricated Formlabs crowns exhibited the lowest marginal gap values, whereas PZCs showed the highest marginal discrepancies. Furthermore, all tested resin crown materials demonstrated marginal gap values within clinically acceptable ranges.

Based on these findings, the evaluated 3D-printed resin crown systems demonstrated favorable marginal adaptation. However, these findings are limited to marginal gap evaluation under the present in vitro conditions. Further in vivo studies assessing long-term clinical performance, mechanical behavior, and biological outcomes are required before definitive clinical recommendations can be made.

## Figures and Tables

**Table 1:** Mean marginal gap values of PZCs and 3D-printed resin crowns.

	Marginal Gap in µm (Mean ± SD)			
	Saremco	Formlabs	Graphy	Prefabricated Zirconia Crowns
**Average**	**118.54 ± 1.58 ^a^**	**113.22 ± 1.94 ^b^**	**134.23 ± 2.16 ^c^**	**163.14 ± 3.95 ^d^**

Data are presented as mean ± SD [95% CI]; n = 10 per group. Different superscript letters indicate statistically significant differences between groups according to the Games–Howell post hoc test (*p* < 0.05).

## Data Availability

The original contributions presented in this study are included in the article. Further inquiries can be directed to the corresponding author.
